# Electrochemotherapy Modulates Mammary Tumor Growth in Rats on a Western Diet Supplemented with Curcumin

**DOI:** 10.3390/biomedicines8110498

**Published:** 2020-11-13

**Authors:** Raji Sundararajan, Lakshya Mittal, Ignacio G. Camarillo

**Affiliations:** 1 School of Engineering Technology, Purdue University, West Lafayette, IN 47907, USA; lmittal@purdue.edu; 2 Department of Biological Sciences, Purdue University, West Lafayette, IN 47907, USA; ignacio@purdue.edu; 3 Purdue Center for Cancer Research, Purdue University, West Lafayette, IN 47907, USA

**Keywords:** curcumin, western diet, breast cancer, rat mammary tumor, N-methyl-N-nitrosourea, electrochemotherapy

## Abstract

In the US, every 12 min, six women are diagnosed with breast cancer and one dies. This highlights a critical need for developing alternate therapies using natural compounds, which are cost effective and with less side effects. Curcumin, the yellow pigment of turmeric has been found to suppress initiation, progression, and metastasis of a variety of tumors. Multiple clinical trials highlight the efficacy of curcumin in treating breast cancer and other diseases. Our in vitro studies have demonstrated that the electrical pulse (EP) application can further enhance the effectiveness of curcumin against breast cancer cells in a therapy called electrochemotherapy (ECT). In a direct extension of these results, we studied the effect of ECT coupled with intratumoral curcumin administration (EP+Cur) on N-methyl-N-nitrosourea (MNU) induced mammary tumors in female Sprague Dawley rats. Beginning at the weaning and throughout the study, rats were fed either western diet (West) or western diet, supplemented with 1% curcumin (W+Cur). Our results showed that EP+Cur treatment led to a reduced growth rate in rats fed with W+Cur diet compared to West diet (57.14% vs. 16.67% in West diet). These results provide a foundation for further studies towards utilizing it in clinical practice.

## 1. Introduction

Breast cancer is the most common cancer of women all over the world. In the US, every 12 min, six women are diagnosed with breast cancer and one dies [1]. Despite several breakthroughs in prevention, control, and the treatment of cancer, millions of patients are afflicted and incurable because of the poor response rate, high cost, and side effects of the commonly administered approved drugs. Thus, there is a critical need for effective alternate therapies using natural compounds, which are not only cost effective, but also have less side effects compared to conventional therapeutics.

Studied in this research is curcumin, the yellow pigment component of natural herb turmeric (constitutes 2–5%), that has been used for over 4000 years in the Indian subcontinent and other Asian countries as a dietary supplement, food preservative, coloring agent, and medicine [2]. In Ayurveda (science of life), turmeric is recommended for various diseases and conditions, such as skin, pulmonary, and gastrointestinal systems, aches, pains, wounds, sprains, and liver disorders [2]. The majority of the bioactivities of turmeric are attributed to curcumin, and it remains the most extensively studied compound of turmeric. The first study on curcumin’s biological activity as an antibacterial agent was reported in 1949 in Nature against staphylococcus aureus, salmonella paratyphi, trichophyton gypseum, and mycobacterium tuberculosis strains [3].

Curcumin has been found to suppress initiation, progression, and metastasis of a variety of tumors, such as breast, cervical, colorectal, bladder, lung, skin, stomach, and oral cancers, by modulating multiple receptors, growth factors, inflammatory cytokines, kinases, enzymes, and transcription factors [2,4]. Curcumin targets several genes and proteins to modulate multiple pathways (NF-κB, PI3K/Akt/mTOR, MAPK, Wnt/β-catenin, Notch, and Hh pathways) and exert its anticancer action [5,6].

The first curcumin clinical trial was published in 1937 in Lancet on human biliary diseases [7]. Due to its excellent characteristics, the research on curcumin has increased significantly over the years, as demonstrated by more than 15,000 research articles (in PubMed) and 236 clinical trials (in ClinicalTrials.gov) with keyword curcumin as of September 2020 [8,9]. Among 49 double-blind placebo-controlled clinical trials of curcumin, 17 recent trials showed efficacy [10].

In cancer patients, most trials of curcumin involve therapy of colorectal and pancreatic cancers. Currently, 6 studies using curcumin as drug or dietary supplement for breast cancer treatment are registered with ClinicalTrials.gov [11]. Oral curcumin reduced the severity of radiation dermatitis in breast cancer patients in a randomized, double-blind, placebo-controlled clinical trial [12]. In a dose escalation trial of docetaxel with curcumin in patients with advanced and metastatic breast cancer, antitumor activity of curcumin and docetaxel combination was demonstrated [13]. A daily intake of curcumin, up to 8 g was found to be safe [13]. In another comparative, randomized, double-blind, placebo-controlled clinical trial, intravenous infusion of curcumin with paclitaxel was superior to paclitaxel with placebo in the treatment of advanced and metastatic breast cancer [14]. These studies highlight the efficacy of curcumin in breast cancer treatment.

To further enhance the effectiveness of curcumin, several approaches for increasing delivery of curcumin have been explored [15]. However, the clinical potential of these formulations is yet to be established. Towards this, electrical pulse (EP) application can be effective to increase the delivery of curcumin in cells by opening cell membrane pores, since an enhanced cytotoxicity (up to 1000-times) can be obtained at lower concentrations of curcumin [16], using this non-surgical procedure, called electrochemotherapy (ECT).

ECT is an efficient modality to treat local tumors, and it has shown promising success in the clinics for the treatment of various cancers, as it is applicable to all histology of cancers [16]. Several clinical studies have demonstrated the efficacy of ECT in treating advanced subcutaneous and cutaneous tumor nodules, including malignant melanoma, squamous cell carcinoma, basal cell carcinoma, keratoacanthoma, and breast cancer and its metastases [16,17,18,19,20,21,22,23].

ECT is effective against mammary tumors in animal models. In a spontaneous mammary tumor mouse model, weekly ECT treatment resulted in partial regression in all 38 tumors, complete remission in 23 tumors, and cure in 3 tumors [24]. In another study, two sessions of ECT treatment of incompletely excised mammary carcinoma in two male pet rats led to complete remission of tumors after 10 and 14 months [25]. Tozon et al. also showed a significant reduction in the volumes of tumor nodules treated with ECT in 2 cats and 1 dog with mammary adenocarcinoma, who were previously treated with surgery [26]. In these studies, the ECT treatment was well tolerated and no major local or systemic side effects were observed after the treatment.

In clinical studies, Campana and colleagues used ECT with bleomycin to treat chest wall recurrence (CWR) of breast cancer after mastectomy in 51 patients of median age 70 year (range 38–88), who experienced tumor progression despite re-irradiation and were refractive to several systemic treatments [27,28,29]. In this study, a median of two ECT courses (range 1–5) were performed to obtain a complete 2-month objective response (OR) in 43.2% of patients (22/51) and a partial response (PR) in 47.5% of patients (24/51) [27]. Rebersek et al. used ECT with cisplatin to treat 12 lesions in six metastatic breast cancer patients, showing OR in all 12 lesions (100%), CR in 4 lesions (33%), and PR in 8 lesions (67%), with more than 50% decrease in the lesion [22]. In another study of ECT to treat breast cancer skin metastasis in 7 patients, an OR of 86% was obtained, with 43% of CR and 43% of PR [30]. In the European Standard Operating Procedures for ECT (ESOPE) project, among 58 breast cancer nodules treated with ECT, 52 (~90%) showed CR and 6 (5%) showed PR [16]. In a large cohort of 119 patients with breast cancer cutaneous metastases from International Network for Sharing Practice on Electrochemotherapy (INSPECT) database from 10 European clinics, the evaluation of response in 90 patients at 2-months following ECT resulted into a CR in 45 patients (50%), PR in 19 (21%), stable disease in 16 (18%), and disease progression in 7 (8%) [31]. These and other studies [32,33,34,35,36,37] demonstrate the clinical efficacy of ECT in treating advanced, recurrent, and metastatic breast cancers, which are refractory to multiple modalities, such as surgery, chemotherapy, and radiation.

ECT is simple, repeatable, highly effective, and safe in various subsets of patients with advanced, recurring, radio- and chemo-resistant tumors. Considering its excellent outcomes, its safety, and affordability, ECT is now recommended for primary skin cancer and cutaneous metastases in national and international guidelines [38,39]. Typically, ECT is used with conventional drugs, such as bleomycin and cisplatin, which come with high cost and severe side-effects on their own. Considering this, our group specializes in ECT for breast cancer treatment using natural compounds and extracts, such as curcumin, and have shown excellent increase in their cytotoxicity against breast cancer cells [40,41,42,43,44,45,46]. We have shown that ECT with curcumin enhances the cytotoxicity of curcumin against breast cancer cells, while causing limited cytotoxicity in non-cancerous epithelial cells [41,42,46].

In a direct extension of these in vitro results, we studied the effect of ECT with intratumoral curcumin (EP+Cur) on *N*-methyl-*N*-nitrosourea (MNU) induced mammary tumors in Sprague Dawley (SD) rats. The SD rat model is a widely recognized model due to its strong histological and genetic resemblance with human breast tumors [47,48,49]. Moreover, due to the beneficial effect of dietary curcumin, we studied if western diet (West) supplemented with curcumin (W+Cur) had any correlation with the outcome of the ECT treatment with curcumin. This study is the first of its kind to use ECT with curcumin on a rodent model to determine the influence of curcumin supplement diet on the efficacy of ECT to treat tumors.

## 2. Materials and Methods

### 2.1. Animals

Forty virgin female Sprague Dawley (SD) rats (Envigo, Indianapolis, IN, USA) were utilized. Upon arrival, all rats were housed in pair with continuous access to water, food, and environmental enrichment. All animal experiments were approved by the Purdue Animal Care and Use Committee. (Protocol: 111000342).

### 2.2. Diets

The SD 21-day weaning rats were randomly divided into 2 groups, Group 1, and Group 2. Group 1 was fed with western (West) diet (D12079B; Research Diets, NJ, USA). The West diet (D12079B, Research Diets, Inc.) contained high fat (40% Kcal) and high carbohydrate (43% Kcal).

Group 2 was fed with western diet supplemented with curcumin (1% by weight) (W+Cur). Here, only curcumin, not turmeric was used.

**Figure 1:**
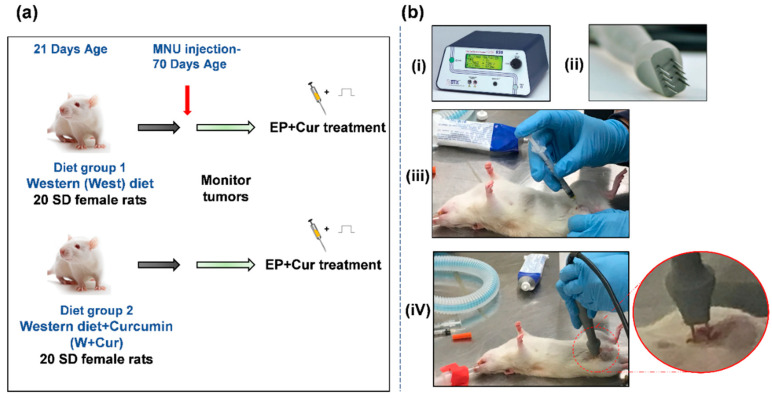
(**a**) Experimental design of the study. (**b**) The electrochemotherapy (ECT) with curcumin treatment: (i) BTX ECM 830 electroporator for applying square wave electrical pulses (EP) (1000 V/cm, 100 µs, 8 pulses at 100 ms interval between pulses). (ii) Needle array electrodes composed of two rows of 4 needles, used for EP application to tumor. (iii) Intratumoral administration of curcumin. (iv) EP application after 1 min of curcumin injection. The zoomed-up Inset shows a typical electrode placement on tumor for EP application.

The W+Cur diet (D17061304) was also prepared by Research Diets, Inc. The curcumin compound (Powder City curcumin 95%) was first weighed to add to the West diet mix at the necessary amount (10 g curcumin per 1 kg West diet) to provide the final diet mixture with 1% *w*/*w* curcumin and then this was incorporated into a premix including all dry ingredients and the color dyes, which allowed to observe the homogeneity of the mixture once the color was evenly distributed. Next, a premix of corn oil and ethoxyquin (antioxidant) was added and then the appropriate amount of butter was added to the entire mixture at the correct level. First, each ingredient was homogeneously mixed and then a small amount of water was added for the purpose of pelleting (around 5–10% of the weight of the diet). Both the diets were forced through a dye and pelleted with a “cold” extrusion process. No heat was intentionally added, but the friction of moving parts during the pelleting process might expose the diet to temperatures around 32.2 °C for less than a minute. After pelleting, the diets were dried for 2 days to remove the water added prior to pelleting. The diets were then irradiated at 37–43 °C for 40–45 min. Table 1 shows the ingredients of two diets. Figure 1a shows the experimental design of the study.

The rats were maintained on their respective diets throughout the study.

### 2.3. Mammary Tumor Induction and Assessment

At 70 days age, all rats were given single injection of *N*-Methyl-*N*-nitrosourea (MNU, NG-17031; Chem Service, Inc., PA, USA) intraperitoneally (i.p.) at 50 mg/kg to induce tumors in the mammary glands, and maintained on their respective diets (Figure 1a). The MNU was delivered via i.p. injection, as this route is the most reliable, rapid, and reproducible for MNU-induced mammary tumorigenesis [50]. MNU was immediately dissolved before treatment and was protected from light. The rats were inspected weekly for mammary tumor incidence, which were palpated by hand and measured with callipers on three axes. When a tumour was first palpated, the date and its location were recorded. The tumor volumes were calculated using Length × Width × Height × π/6 in mm^3^.

### 2.4. Electrochemotherapy Treatment

When the tumors were about 1 cm, rats were treated with curcumin (C1386; Sigma Aldrich, MO, USA) and electrical pulses (EP). Figure 1b shows the equipment and procedure used for the treatment. Curcumin was first dissolved into DMSO (BP231-100; Fisher scientific, MA, USA) and was diluted further in saline to make 200 µM concentration for injections. First, curcumin injections (100 µL) were administered intratumorally. After 1 min of curcumin injection, multiple EP (1000 V/cm, 100 µs, 8 pulses at 100 ms interval between pulses) were administered using needle array electrodes (Leroy Biotech, France) composed of two rows of 4 needles (15 mm long, 2.9 mm gap between needle tips and 5.9 mm gap between rows) to cover the entire tumor area. The BTX ECM 830 electroporator (BTX, MA, USA) was used for generating EP. The rats were anesthetised during the procedure. When the mammary tumors reached 2.5 cm in any one dimension, rats were euthanized.

### 2.5. Quantitative Characterization of Mammary Tumor Growth

Tumor volumes post treatments were quantitatively compared to the tumor volume before start of the treatment (at day 0), using relative tumor volumes and specific growth rate (SGR). The relative tumor volumes were calculated using Equation (1).

(1) $$Relative\;tumor\;volume=\frac{V_{1}}{V_{0}}.$$

The SGR calculations were done using Equation (2), as described previously [51].

(2) SGR=ln(V1V0)/(t1−t0),

where *V*_0_ and *V*_1_ are the tumor volumes before treatment (*t*_0_) and tumor volume at a particular day (day 7 or 14) after the treatment (*t*_1_), respectively. For quantitative characterization of tumor growth in rats with missing tumor volume measurements at days 0, 7, or 14, the tumor volumes at these time points were estimated between two known values manually in GraphPad Prism.

### 2.6. Statistical Analysis

Student’s t-test was used to compare groups. Tumor onset data was analyzed using the Kaplan–Meier method, followed by log rank test using chi square statistics in STATA. Cox proportional hazard regression model was also run on the tumor onset survival data to estimate the ratio of hazard rate in two diet groups. Correlation between tumor size at treatment and tumor responses was assessed using Fisher’s exact test (two tail) in JMP software.

A repeated measure ANOVA analysis was performed in JMP to compare the growth rate of all the tumors (including second and third tumors) before treatment (before day 0) for the rats who responded to the treatment to the ones who did not show a response in Cur diet with EP+Cur treatment group.

For all tests, values were considered significant at *p* < 0.05. Results are expressed as mean ± standard error (SE).

## 3. Results

### 3.1. Rat Postnatal Development

**Figure 2:**
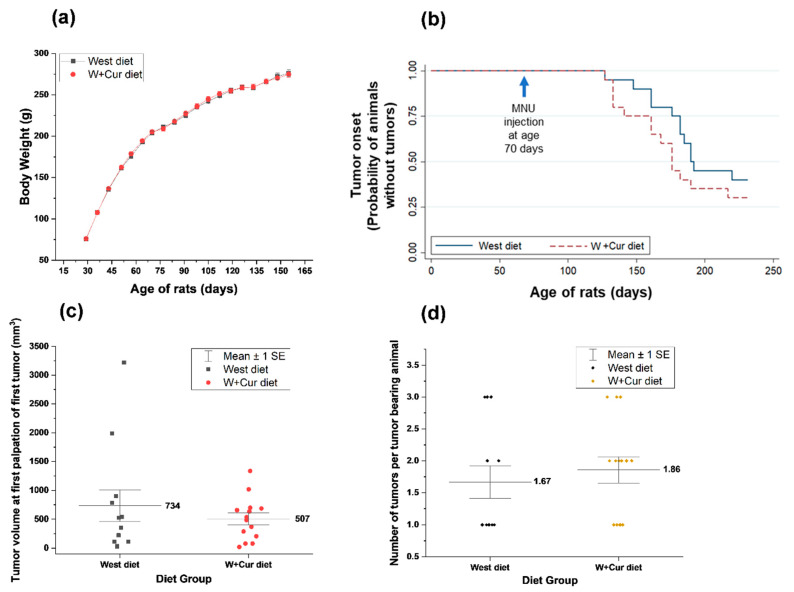
(**a**) The body weights of rats in both western (West) and western supplemented with 1% curcumin (W+Cur) diet groups. (**b**) Tumor onset analysis using Kaplan–Meier method. *Y*-axis shows the probability of animals without tumor incidence. (**c**) Volume of the first tumor at inception (earliest palpable tumor) for both the groups. (**d**) Number of tumors per tumor bearing animal.

Administration of MNU did not induce any acute toxicity in treated rats. Figure 2a shows the average body weight of rats for West diet and W+Cur diet groups over days. No significant difference was observed in the body weight between two groups at any time point.

### 3.2. Mammary Tumor Incidence and Multiplicity

The first palpable mammary tumors appeared during the 9th week (57days) of the MNU application in both diet groups (rat age 127 days). At this time only one rat from each group developed a palpable tumor. As time progressed, the incidence of mammary tumor increased. At the end of the study, the overall incidence of palpable mammary tumors was 60% (12/20 rats) in West diet, while it was 70% (14/20 rats) in W+Cur diet, indicating similar tumor incidence in both groups. The median time to tumor onset were 182 days of age in West diet, while it was 164.5 days in W+Cur diet.

The Kaplan–Meier method was used to study the mammary tumor latency in these two diet groups. Figure 2b shows the probability of animals without tumor incidence from Kaplan–Meier method. To test if the rats in one diet group have a longer time to first occurrence of tumor than those in the other, we conducted a log rank test using chi square statistics. The *p*-value from chi square test was 0.2748, indicating that the survival curves for two groups are not significantly different from each other. Cox proportional hazard regression model was also run on the tumor onset survival data to estimate the ratio of hazard rate in two diet groups. The hazard ratio was 1.51, with a 95% confidence interval of (0.69–3.28) and *p*-value of 0.2927, indicating that the tumor onset for the rats in two diet groups are not significantly different from each other.

We also compared the volume of the first detectable tumor at palpation for both the groups. These results are shown in Figure 2c. Mean volume at tumor onset was 734 mm^3^ for West diet, while it was 507 mm^3^ for W+Cur diet. Even though the mean tumor volume at first palpation for W+Cur diet group was lower, it was not significantly different from West diet group. Figure 2d shows the tumor multiplicity per tumor bearing animal, which was 1.67 for West diet group and 1.86 W+Cur diet, not significantly different from each other.

### 3.3. Mammary Tumor Growth before Treatment

**Figure 3:**
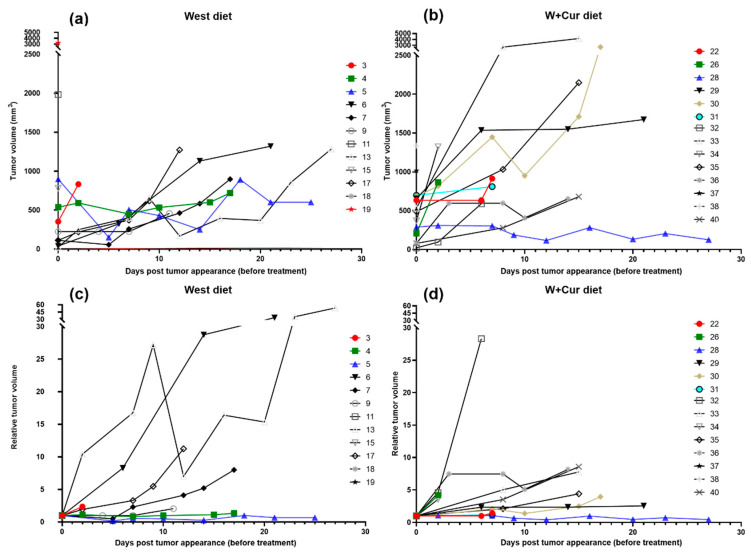
Individual tumor growth curves showing absolute (**a**,**b**) and relative (**c**,**d**) tumor volumes over days post tumor appearance but before treatment start: (**a**,**c**) West diet, (**b**,**d**) W+Cur diet. The tumor volumes were normalized with the tumor volume at inception to calculate the relative tumor volume.

Further, we studied the relative change in the volume of the first tumor post appearance for both diet groups, but before the start of treatment. Figure 3a,b show the absolute tumor volumes for individual rats in West and W+Cur diet, respectively. The tumor volumes were also normalized with the tumor volume at inception to calculate the relative tumor volume, as shown in Figure 3c,d. A considerable variability was observed in the tumor growth of individual rats within both diet groups. Several rats in both groups showed exponential tumor growth. The tumor growth was similar for both diet groups.

Together these results highlight that the W+Cur diet did not impact postnatal development, tumor incidence, latency, multiplicity, and growth rates.

### 3.4. Mammary Tumor Growth after Treatment

**Figure 4:**
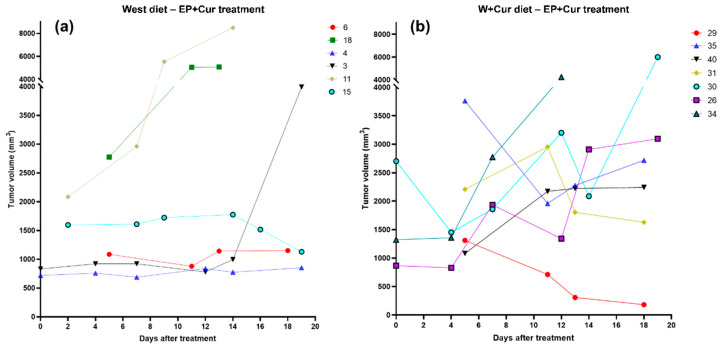
Individual tumor growth curves showing absolute tumor volumes over days after treatment: (**a**) West diet with ECT with intratumoral curcumin (EP+Cur) treatment, (**b**) W+Cur diet with EP+Cur treatment.

Tumor volumes were measured after treatment with EP+Cur for rats in West and W+Cur diet (Figure 4a,b). To quantitively characterize the tumor growth after treatment in both diet groups, relative tumor volumes and SGR were calculated at days 7 and 14 following treatment, with respect to pre-treatment volumes (on day 0). These results are shown in Table 2 and Table 3 for West and W+Cur diet, respectively. Relative tumors and SGR were also measured in second and third tumors appeared in the treated rats (Table 4). A negative SGR (i.e., reduction in tumor volume) at day 14 compared to day 0 was considered to be a tumor response. When rats on West diet were treated with EP+Cur, only 16.67% (1/6) showed a tumor response in at least one tumor nodule. On the other hand, 57.14% (4/7) rats on W+Cur diet showed tumor response in at least one nodule upon EP+Cur treatment.

Further, we also determined a correlation between tumor size at treatment on day 0 and tumor response for rats on W+Cur diet and EP+Cur treatment. Fisher’s exact test (two tail) revealed that there was a significant (*p*-value = 0.0286) correlation between tumor response and tumor size (>1500 mm^3^) for W+Cur diet with EP+Cur treatment.

Tumors that showed a lack of response to treatment on W+Cur diet were not associated with differences in pre-treatment growth rate. To support this, we compared the growth rate of all the tumors (including second and third tumors) before treatment (before day 0) for the rats. For example, in rats 31 and 40 which did not respond to the treatment, tumor sizes were 50% and 61% at 7 days before treatment compared to the day of treatment. In comparison, at 7 days before treatment, the sizes were 52% and 35% for rats 35 and 30, which responded to the treatment. This indicates that the tumor growth rate before treatment was similar in tumors irrespective of the response to EP+Cur treatment. This was confirmed by the repeated measure ANOVA analysis on all tumors, which showed that there was no significant difference between these two groups (*p*-value = 0.9314).

The extent of visual necrosis in the tumor tissues was also analyzed in the tumor tissues collected post euthanasia. Table 2, Table 3 and Table 4 show the percentage of necrotic tumor tissue. There was no correlation between the percentage of necrosis and tumor response for both treatment groups, indicating that the levels of necrosis did not influence the tumor response.

Together, these results suggest that EP+Cur treatment results in a better response, when the rats are fed W+Cur diet, with larger nodules showing significantly higher response rates compared to smaller nodules, despite same level of growth in both, before treatment.

## 4. Discussion

In the present study, we investigated the effect of electrochemotherapy with curcumin on the spontaneous mammary tumor growth in female SD rats on West or W+Cur diet. The SD rats-MNU system used in this study is a widely accepted model of human breast cancer [47,48,49]. The mammary tumor histology in this model resembles strongly to that of human breast tumors [48,49]. Similar to humans, rat model mammary tumors are primarily ductal in origin, as compared to mouse lesions, which are mainly alveolar [52]. The MNU induced mammary tumors remains one of the most frequently used models to study mammary tumor carcinogenesis and treatment [48,52,53]. The MNU model offers several advantages, including easy application without needing irradiation or metabolic activation for tumorigenesis, reliability of tumor induction, organ specificity, and ductal origin of the tumors [50,52].

We observed that the final incidence of palpable mammary carcinoma was 60% in West diet and 70% in W+Cur diet after MNU administration. This is in agreement with previous SD rat studies, where 60–76% of the incidences of palpable mammary carcinomas were observed upon MNU administration [52,53]. In our study, the first palpable mammary tumors were observed during the 9th week (57 days) of the MNU application in both diet groups (rat age 127 days), which matches extremely well with previous observation, where palpable tumors appeared 9 weeks after the first application of MNU in SD rats [47]. These observations are in line with the existing literature and establish the validity of the model to demonstrate the efficacy of ECT with curcumin.

We did not observe any significant difference between West and W+Cur diet groups in tumor onset, incidence, multiplicity, and growth. These results were expected, as we intentionally added a low concentration of curcumin in West diet (1% *w*/*w*) to avoid confounding in our study, as, a delay in tumor onset and growth due to curcumin diet alone could impact tumor treatment response. This correlates with previous studies, where a higher (2% *w*/*w*) concentration of dietary curcumin did not reduce the tumor incidence, latency, and multiplicity in carcinogen induced mammary tumorigenesis [54,55].

Herein we demonstrate that EP+Cur treatment resulted in a better tumor response, when the rats were fed with W+Cur diet (57.14% response vs. 16.67% response in West diet). Further, we showed that the larger tumor nodules (>1500 mm^3^) within Cur diet were more likely to exhibit significantly higher response rates compared to smaller nodules upon EP+Cur treatment, despite same level of pre-treatment growth in both.

Regarding curcumin’s actions, despite the low bioavailability of curcumin, its metabolites could positively influence the effect of EP+Cur treatment we observed in this study. Curcumin is rapidly and efficiently transformed into metabolites, which reduces the bioavailability of parent compound curcumin upon entering the body [56]. However, in circulation, curcumin undergoes conjugation, including glucuronidation and sulfation in tissues, mostly in the liver [57]. These conjugates are now known to exert antioxidative, anti-inflammatory, and anti-cancer effects [58]. In addition, we and others have quantitively shown the enhanced uptake and cytotoxicity effects of curcumin when delivered with electrical pulses [41,42,46,59].

In this study, we used 1% curcumin concentration in the diet. However, it will be of interest to further study the effect of various curcumin concentrations in diet on the outcome of EP+Cur. Previously, a study showed that a 2% *w*/*w* dietary curcumin with paclitaxel decreased the breast cancer metastasis to lung by downregulating paclitaxel-induced NF-κB–regulated gene products [60]. Considering this, we hypothesize that a higher concentration of curcumin in diet (2% or more) may further enhance the effects of EP+Cur treatment.

This is the first study, demonstrating the enhanced effects of ECT with curcumin on rat mammary tumors with W+Cur diet. Although, in this study the tumor initiation and progression under these novel conditions were studied, the limitation of this study is that a detailed investigation on histopathological phenotype of the treated tumors with respect to the controls was not included. Future work will investigate histopathological features and immunohistochemistry analyses of apoptosis, cell proliferation, and angiogenesis.

However, these results do provide a foundation for further studies, such as characterizing the association between W+Cur diet and the effectiveness of EP+Cur. Previous studies have reported that curcumin exhibits a diverse array of metabolic, cellular, and molecular activities including potent anti-inflammatory actions due to its ability to target pro-inflammatory pathways, such as NF-κB, MAPK, and JAK/STAT signaling pathways [4,61]. The chronic inflammation promotes cancer cell growth through direct or indirect interactions between inflammatory cells and cancer cells, including release of growth factors, removal of growth suppressors, and enhanced resistance to cell death [4]. The inflammatory tumor microenvironment is also linked with the metabolic reprogramming to aerobic glycolysis (Warburg effect). The pro-inflammatory cytokine TNF-α induces aerobic glycolysis, lactate export, and expression of the glucose transporter 1 (GLUT1), which is reversed by dietary curcumin [61,62]. In connection with these documented actions of curcumin, our group has previously demonstrated that the EP+Cur treatment targeted glycolysis with reduced glucose uptake and intracellular lactate in MDA-MB-231 breast cancer cells to trigger apoptotic cell death [42]. Thus, a possible explanation of the better tumor response observed in Cur diet group upon EP+Cur treatment in the present study could be the result of the combination of these long-term and related mechanisms. In future, it will be worthwhile investigating other promising plant derived compounds, such as lycopene and resveratrol [63], which could have positive influence on better response of EP+Cur.

## Figures and Tables

**Table 1:** The ingredients of western (West) diet and western diet supplemented with 1% *w*/*w* curcumin (W+Cur).

	Diet	West Diet		W+Cur Diet	
Ingredient					
		gm	kcal	gm	kcal
Casein		195	780	195	780
DL-Methionine		3	12	3	12
Corn Starch		50	200	50	200
Maltodextrin 10		100	400	100	400
Sucrose		341	1364	341	1364
Cellulose, BW200		50	0	50	0
Milk Fat, Anhydrous		200	1800	200	1800
Corn Oil		10	90	10	90
Soybean Oil		0	0	0	0
Ethoxyquin		0.04	0	0.04	0
Mineral Mix S10001		35	0	35	0
Calcium Carbonate		4	0	4	0
Vitamin Mix V10001		10	40	10	40
Choline Bitartrate		2	0	2	0
Cholesterol		1.5	0	1.5	0
**Curcumin, 95%**		0	0	**10.65**	0
FD&C Red Dye #40		0	0	0.025	0
FD&C Yellow Dye #5		0	0	0.025	0
Total		1001.5	4686	1012.2	4686
Curcumin (g/kg)		0		10	

**Table 2:** Quantitative characterization of tumor growth for rats on West diet with EP+Cur treatment. The relative tumor volumes and specific growth rate (SGR) were calculated for days 7 and 14 after treatment, compared to the tumor volume before start of the treatment (at day 0).

**Absolute Tumor Volumes**							
	**Rat**	**6**	**4**	**3**	**11**	**15**	**18**
**Day**							
**0**		1259	720	833	2020	1067	1184
**7**		1028	690	922	2964	1612	3561
**14**		1149	777	996	8487	1777	-
**Necrosis**		15%	<5%	10%	20%	50%	-
**Relative Tumor Volumes**							
	**Rat**	**6**	**4**	**3**	**11**	**15**	**18**
**Day**							
**0**		1.00	1.00	1.00	1.00	1.00	1.00
**7**		0.82	0.96	1.11	1.47	1.51	3.01
**14**		0.91	1.08	1.20	4.20	1.67	-
**Specific Growth Rate (SGR)**							
	**Rat**	**6**	**4**	**3**	**11**	**15**	**18**
**Day**							
**7**		−0.029	−0.006	0.015	0.055	0.059	0.157
**14**		−0.007	0.005	0.013	0.103	0.036	-

**Table 3:** Quantitative characterization of tumor growth for rats on W+Cur diet with EP+Cur treatment. The relative tumor volumes and SGR were calculated for days 7 and 14 after treatment, compared to the tumor volume before start of the treatment (at day 0).

**Absolute Tumor Volumes**								
	**Rat**	**29**	**30**	**35**	**26**	**31**	**34**	**40**
**Day**								
**0**		1580	2702	2630	866	1223	1323	808
**7**		1118	1859	3165	1935	2472	2773	1459
**14**		294	2087	2378	2909	1780	-	2236
**Necrosis**		10%	5%	25%	15%	30%	30%	15%
**Relative Tumor Volumes**								
	**Rat**	**29**	**30**	**35**	**26**	**31**	**34**	**40**
**Day**								
**0**		1.00	1.00	1.00	1.00	1.00	1.00	1.00
**7**		0.71	0.69	1.20	2.23	2.02	2.10	1.81
**14**		0.19	0.77	0.90	3.36	1.45	-	2.77
**Specific Growth Rate (SGR)**								
	**Rat**	**29**	**30**	**35**	**26**	**31**	**34**	**40**
**Day**								
**7**		−0.049	−0.053	0.026	0.115	0.101	0.106	0.084
**14**		−0.120	−0.018	−0.007	0.087	0.027	-	0.073

**Table 4:** Quantitative characterization of growth of second (T2) and third (T3) tumors only, after treatment with EP+Cur for both West and W+Cur diet. The relative tumor volumes and SGR were calculated for days 7 and 14 after treatment, compared to the tumor volume before start of the treatment (at day 0).

**Absolute Tumor Volumes**								
		**West Diet–EP+Cur**		**W+Cur Diet–EP+Cur**				
	**Rat**	**6–T2**	**6–T3**	**29–T2**	**35–T2**	**35–T3**	**31–T2**	**31–T3**
**Day**								
**0**		780	514	2331	1176	992	982	1360
**7**		879	520	1633	1391	1134	168	1386
**14**		604	193	-	-	-	204	1981
**Necrosis**		5%	5%	30%	5%	5%	10%	10%
**Relative Tumor Volumes**								
	**Rat**	**6–T2**	**6–T3**	**29–T2**	**35–T2**	**35–T3**	**31–T2**	**31–T3**
**Day**								
**0**		1.00	1.00	1.00	1.00	1.00	1.00	1.00
**7**		1.13	1.01	0.70	1.18	1.14	0.17	1.02
**14**		0.77	0.38	-	-	-	0.21	1.46
**Specific Growth Rate (SGR)**								
	**Rat**	**6–T2**	**6–T3**	**29–T2**	**35–T2**	**35–T3**	**31–T2**	**31–T3**
**Day**								
**7**		0.017	0.002	−0.051	0.024	0.019	−0.252	0.003
**14**		−0.018	−0.070	-	-	-	−0.112	0.027
